# A Comparison Study: Possible Bias in Troponin I Measurement Obtained with a Point of Care Testing and a Central Laboratory Analyzers Employing Different Biological Matrices and Anticoagulants

**DOI:** 10.3390/diagnostics14141482

**Published:** 2024-07-10

**Authors:** Antonino Sammartano, Ruggero Buonocore, Roberto Fiorini, Elisabetta Dieci, Anna Di Franco, Bruna Di Stasi, Giovanni Tortorella, Luigi Ippolito

**Affiliations:** 1 U.O. Clinical Pathology, Medical and Diagnostics Department P.O. Fidenza, Azienda AUSL of Parma, 43125 Parma, Italy; lippolito@ausl.pr.it; 2 U.O. Biochemical, Department of Clinical Patology, Ospedale Guglielmo da Saliceto of Piacenza, 29121 Piacenza, Italy; r.buonocore@ausl.pc.it (R.B.); e.dieci@ausl.pc.it (E.D.); a.difranco@ausl.pc.it (A.D.F.); b.distasi@ausl.pc.it (B.D.S.); 3 Emergency Department, Vaio Hospital, Azienda AUSL of Parma, 43125 Parma, Italy; 4 Cardiology Unit, Medical and Diagnostics Department P.O. Fidenza, Azienda AUSL of Parma, 43125 Parma, Italy

**Keywords:** high-sensitivity, point of care, troponin, myocardial injury

## Abstract

Background: high-sensitive cardiac TroponinI (hs-cTnI) is widely used for diagnosis of acute coronary syndromes. The latest recommendation for hs-cTnI determination is the protocol 0–1 h finalized to improve the rule out accuracy of the test. A Point of Care Testing able to guarantee these performances could be very useful due to reducing the turnaround time and ruling out patients suspected of ACS, especially by using biological matrices that are not required for centrifuge. The aim of our work is to compare the results for hs-cTnI obtained using different biological matrices and anticoagulants, obtained between Atellica^®^ VTLi hs-cTnI POCT and Access AccuTnI+3 DxI800 performances, in order to establish a possible bias derived directly from these pre-analytical conditions. Methods: Li-heparinized pool samples were primary employ for hs-cTnI with Atellica^®^ VTLi as whole blood, then centrifuged and tested on Atellica^®^ VTLi and DxI800. K_3_EDTA pool samples were centrifuged and measured on DxI800 too. A comparison of methods was performed according to CLSI_EP-09A2 protocol. Constant and proportional errors were investigated with Deming regression. Bias between methods was evaluated with the Bland Altman test. Results: comparing whole blood lithium heparin results obtained with Atellica versus lithium heparin and K_3_EDTA plasma tested on DxI 800, the Deming regression revealed a proportional error, whereas in both cases Bland Altman highlighted a minimal underestimation. A similar performance was revealed when considering plasma lithium heparin tested on Atellica versus lithium heparin and K_3_EDTA plasma obtained with DxI800, confirming the same underestimation. Considering values close to the cut off, no significant differences were found. Conclusions: in the laboratory, the estimation of the bias of two different analyzers is pivotal. Once more this is crucial when different biological matrices and anticoagulants are employed for the analysis. Our study demonstrates that no significant differences among the two matrices are present when comparing Atellica and DxI800 performances.

## 1. Introduction

A high-sensitivity cardiac troponin (hs-cTn) must meet the criteria established by the AACC and IFCC Committee for Clinical Application of Cardiac Biomarkers: first, the imprecision at the cardiac troponin (cTn) concentration of the lowest sex-specific 99th percentile upper reference limit (URL) must be ≤10% CV; second, the measured hs-cTn concentrations in ≥50% of healthy males and females must exceed the assay’s limit of detection (LoD) [1]. Although different methods for high automated platforms present in Central Laboratory (CL) are available and fulfill these criteria, many Point of Care Testing (POCT) lack of these criteria. Elevation in hs-cTn can occur even in cases of Takotsubo syndrome, pericarditis, or major chest trauma, and the release of cTn could be in an acute or in a chronic manner. Thus, in 2020 the European Society of Cardiology (ESC) updated the guidelines for the management of acute coronary syndromes (ACS) in patients presenting without persistent ST-segment where an acute release of cTn is always present. It reported an interesting statement about POCT technologies for cTn which highlights that the majority of currently used POCT cannot be considered high-sensitivity assays [2,3]. Although POCT ensure a short turnaround time (TAT), user-friendly operator conditions, and a very reduced size, for their poor accuracy and imprecision they were considered inadequate for accelerated diagnostic protocol (0/1-h algorithm blood draw) recommend by ESC guidelines and cannot guarantee the fast ruling out of patients [2].

Many manufacturers and colleagues have tried to implemented the POCT cTn sensitivity. Vafaie et al. [4] investigated whether the additional measurement of copeptin, the c-terminal part of the vasopressin prohormone using an ultrasensitive assay, could improve diagnostic performances of AQT90 Flex Radiometer (Radiometer Medical ApS, Brønshøj, Denmark) and Cobas h232 POC-System (Cobas), comparing these results with hs-cTn measured in the CL with Cobas E411 (Roche). They reported that copeptin increased sensitivity of Cobas from 67.9% (95% CI: 0.506; 0.852) to 89.3% (95% CI: 0.778; 1.007) and Radiometer from 71.4% (95% CI: 0.547; 0.882) to 85.7% (95% CI: 0.728; 0.987), achieving the sensitivity of hsTnT alone at admission of 85.7% (95% CI: 0.728; 0.987) [4].

Soresen and Boeddinhaus compared two different POCT hs-cTn analyzers against high-sensitivity assays Central Laboratory methods using the 0/1 h and 0/3 h algorithms in two large cohorts of patients, demonstrating a high diagnostic accuracy in patients with suspected acute myocardial infarction (AMI) and a clinical performance comparable to that of the best-validated Central Laboratory assays [5,6,7].

In 2021, Apple et al. validated the previews criteria mentioned for hs-cTnI of Siemens point of care (POC) Atellica^®^ VTLi hs-cTnI immunoassay. Using heparinized plasma from the AACC universal sample bank (USB), they calculated the 99th percentile URL values for males and females (n = 693, M = 363, F = 330): overall, 23 ng/L [90% confidence interval (CI) 20–32 ng/L]; males, 27 ng/L (90% CI 21–37 ng/L); females, 18 ng/L (90% CI 9–78 ng/L). Moreover, the percentage of patients having a concentration of cTn greater than the LoD was 83.7% (M = 87.3%, F = 79.7%). Apple shows that POC Atellica^®^ VTLi hs-cTnI meets the criteria recommended for high-sensitivity definition by ESC [5,8].

Regarding POCT Atellica^®^ VTLi hs-cTnI, a recent work by Christenson et al. describes a “Roadmap” for the validation and characterization of hs-cTn in lithium heparin plasma and whole blood matrices. This analysis showed excellent agreement between these two matrices in the ACS diagnosis by using POCT assay. The authors reported that a modest positive bias of 6.3% and 3.8% in the relatively low range of ≤50 ng/L and higher range 50 ng/L, respectively, was present, thus discouraging the switching of matrices during serial sampling [9].

The aim of our work is to compare possible differences in hs-cTnI determination using different matrices, by comparing the results obtained with POCT Atellica^®^ VTLi hsTnI and Access AccuTnI+3 applied on DxI 800 (Beckman Coulter, Brea, CA, USA), using whole blood pools lithium heparin (WBLH), plasma lithium heparin (LHP), and plasma K_3_EDTA (PEDTA), as they are normally employed in our laboratory routine workflow.

## 2. Materials and Methods

The comparison regards a pool of 43 samples, which were derived from anonymous leftover patient tubes. 3 samples were discarded due to values above the POCT linearity (>1250 ng/L). The analysis was performed using the remaining 40 samples. All specimens were obtained as residue material of the analysis directly requested from the wards in order to investigate clinical chemistry profiles, complete blood count, or potential heart damage. Hs-TnI concentration varies from 1.90 to 23,666 ng/L.

Li-heparinized whole blood pools were measured with the Atellica VTLi analyzer POCT, then centrifuged (5 min, 3000× *g*) and tested once more on POCT. Plasma K3EDTA and plasma Li-heparinized pools were obtained after centrifugation (5 min, 3000× *g*) and performed with hs-troponin I Beckman Coulter DxI 800 for troponin I determination. This evaluation has been performed according to the CLSI EP09-A3 guideline [10].

### 2.1. Access AccuTnI+3 Troponin I

Hs-cTnI is a chemiluminescence immunoassay performed on Beckman Coulter DxI 800^®^ which employs paramagnetic beads for the quantitative determination of cardiac troponin I (cTnI) ensuring high-sensitivity performance. The methods include the possibility to use serum and plasma Li-heparin, or plasma EDTA as well. The assay exploits a sandwich immune-enzymatic method with a monoclonal antibody specific for cTnI conjugated with an alkaline phosphatase and a dedicated surfactant buffer as solid phase. After the incubation time, paramagnetic beads covered with anti-cTnI monoclonal antibodies are added to the reaction solution. The cTnI, if present, binds the anti-cTnI on solid phase whereas the anti-cTnI conjugated with alkaline phosphatase reacts with the different antigens sites presented by the cTnI. After a second incubation, compounds bound to paramagnetic beads are held in the magnetic field, while the unbound are washed away. Finally, a luminescent substrate, Lumi-Phos 530, is added and the light intensity generated is proportional to the cTnI concentration presents in the sample. Access AccuTnI+3 troponin I uses 55 μL of samples and takes an average time of 20 min [11].

### 2.2. Siemens POC Atellica ^®^ VTLi hs-cTnI

The POCT method called “Siemens POC Atellica^®^ VTLi hs-cTnI” adopts an innovative system that uses Magnotech^®^ type biosensors to separate the cTnI fraction, bound by antibodies to magnetic beads, from the free fraction, and detects the signal using the imaging technique called Frustrated Total Internal Reflection (FTIR) (30). This hs-cTnI POCT system uses a drop (about 30 μL) of whole blood or heparinized plasma, which is introduced into the reaction chamber. Red blood cells are retained by a specific membrane. After the antigen–antibody reaction, which takes place on the sensor surface, the unbound antigen is rapidly removed by a magnetic field oriented in such a way as to rapidly remove the unbound fraction from the sensor surface (i.e., the Magnotech^®^ technique). The reported TAT is <8 min, and the LoD declared by manufacturer is 1.2 ng/L for the heparinized plasma sample and 1.6 ng/L for whole blood EDTA [12].

### 2.3. Method Comparison and Statistics

Comparison of methods was performed according to CLSI EP-09A2 protocol. Constant and proportional errors were investigated with Deming regression. Atellica CV for whole blood K_3_EDTA and whole lithium heparin were, respectively, 6.01% and 7.32%, whereas CV of Beckman Coulter DxI 800 was calculated as 4.16% for both lithium heparin and K_3_EDTA plasma. Bias between methods was evaluated with the Bland Altman test. Statistical analyses were performed with MedCalc^®^ Statistical Software version 20.216 (MedCalc Software Ltd., Ostend, Belgium; https://www.medcalc.org; 2023). No outlier value has been found.

## 3. Results

Table 1 lists the analytical characteristics of troponin assays.

**Figure 1:**
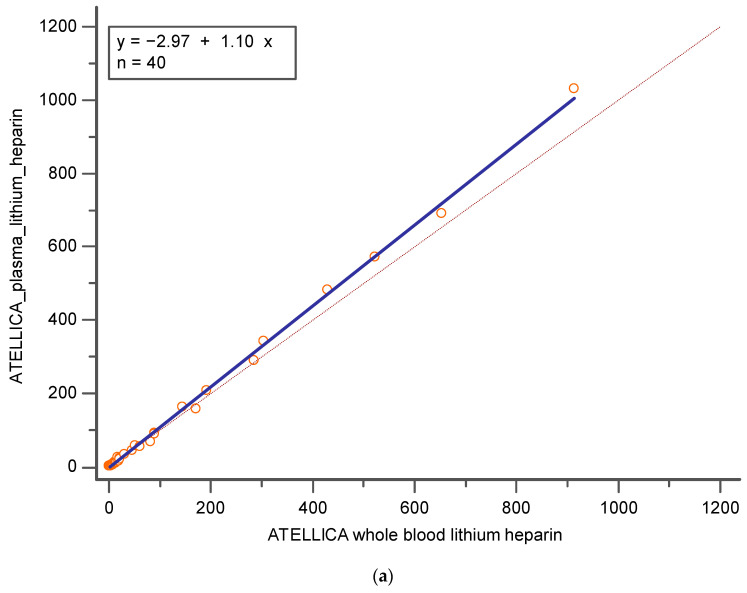

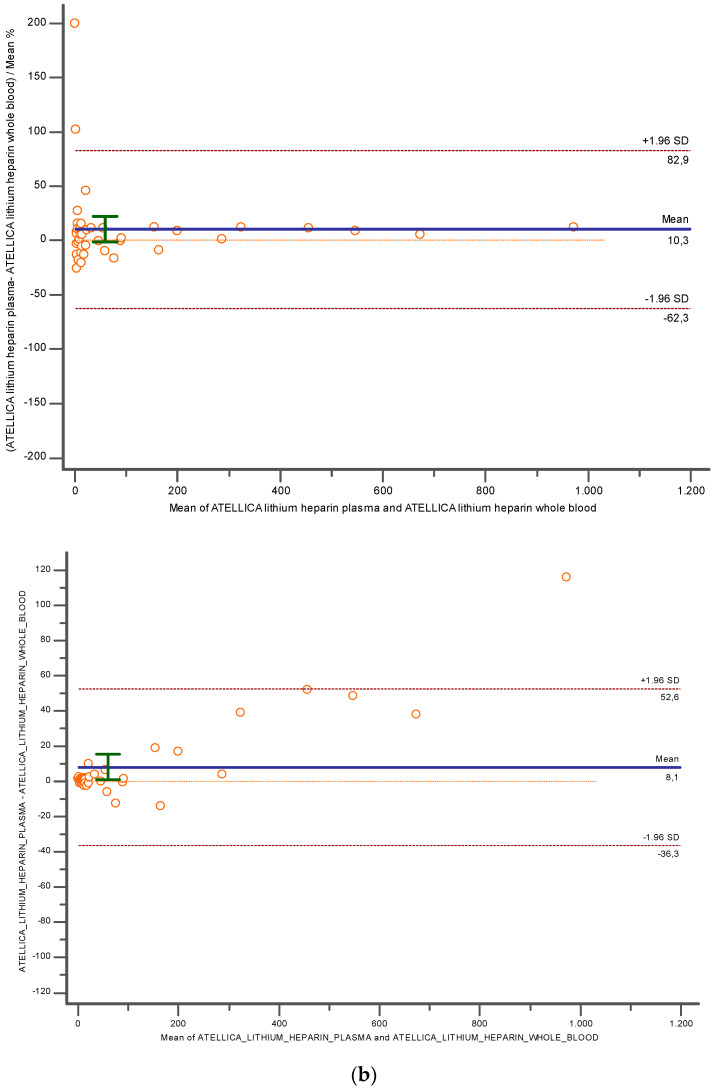
Represents (**a**) Deming regression and (**b**) Bland Altman % between whole blood lithium heparin and plasma lithium heparin, both investigated with Atellica. In blue is reported the line of the equation.

Figure 1a represents Deming regression between whole blood pools lithium heparin and plasma pools lithium heparin, both investigated with Atellica. A good Pearson correlation coefficient of 0.99 (CI = 95%; [0.9981–0.9995]); no constant and a small proportional error were present (y = − 2.97 + 1.10x; slope CI = 95%, [1.0512–1.1561]; intercept CI = 95% [−6.6248–0.6782]). Moreover, no significant differences were highlighted with the Bland Altman test (Figure 1b).

**Figure 2:**
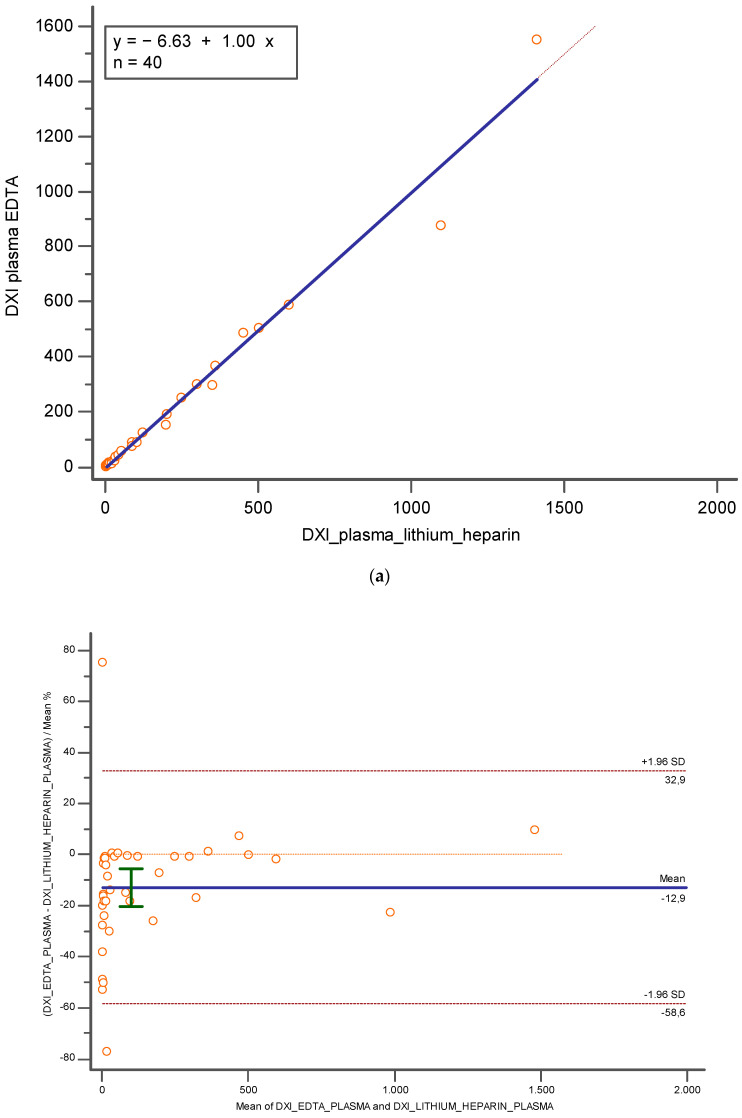

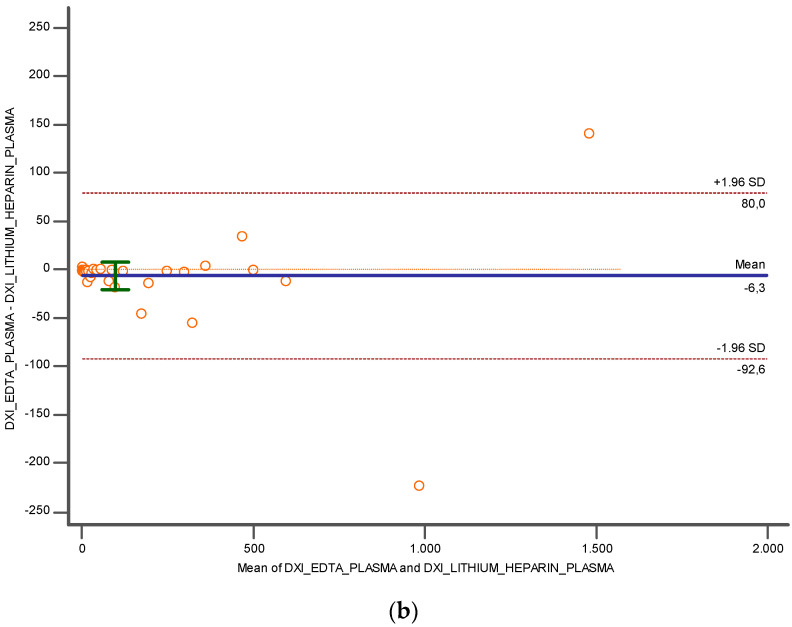
Represents (**a**) Deming regression and (**b**) Bland Altman % between plasma lithium heparin and plasma K3EDTA, both performed on Beckman Coulter DxI 800. In blue is reported the line of the equation.

Figure 2a,b consider the data for plasma lithium heparin and plasma K_3_EDTA, both performed on Beckman Coulter DxI 800. An excellent concordance has been verified too (Pearson correlation coefficient = 0.99, CI = 95%; [0.9795 to 0.9943]), demonstrating no constant and proportional error (y = 6.637 + 1.002x; slope CI = 95%, [0.7414 to 1.2627]; intercept CI = 95% [−29.8224 to 16.5493]) and the absence of any significant bias (Figure 2a,b).

**Figure 3:**
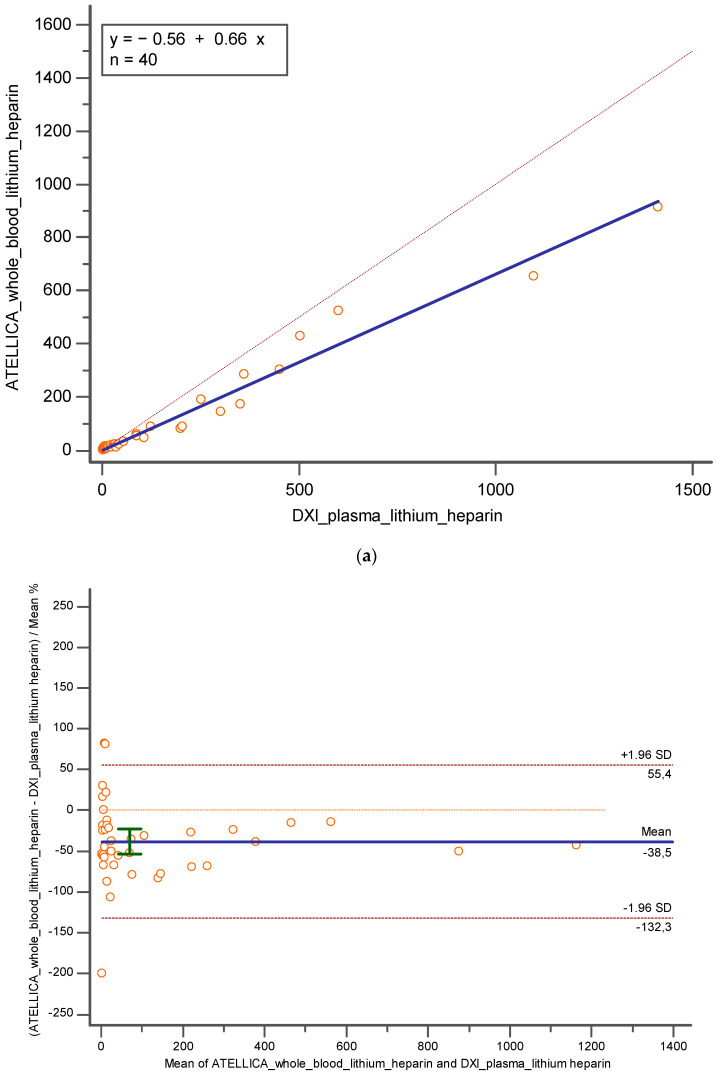

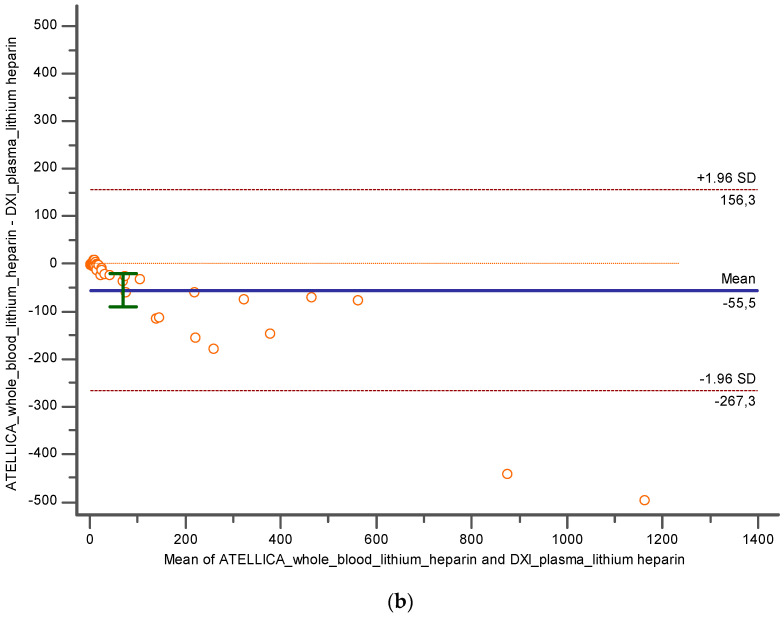
Represents (**a**) Deming regression and (**b**) Bland Altman % between whole blood lithium heparin on Atellica and lithium heparin plasma tested on DxI 800. In blue is reported the line of the equation.

On the other hand, when comparing whole blood lithium heparin results obtained on Atellica and lithium heparin plasma tested on DxI 800, despite the Pearson correlation coefficient of 0.98 (CI = 95%; [0.9719 to 0.9922], the Deming regression revealed a proportional error (y = −0.5622 + 0.6625x; slope CI = 95%, [0.5885 to 0.7365]; intercept CI = 95% [−7.5769–6.4524]) and the Bland Altman highlighted an underestimation of −38.5% (CI = 95% [ −53.7899 to −23.1549]; *p* < 0.001) and −55.5 (CI = 95% [−90.06 to 20.95]; *p* < 0.05) (Figure 3a,b).

**Figure 4:**
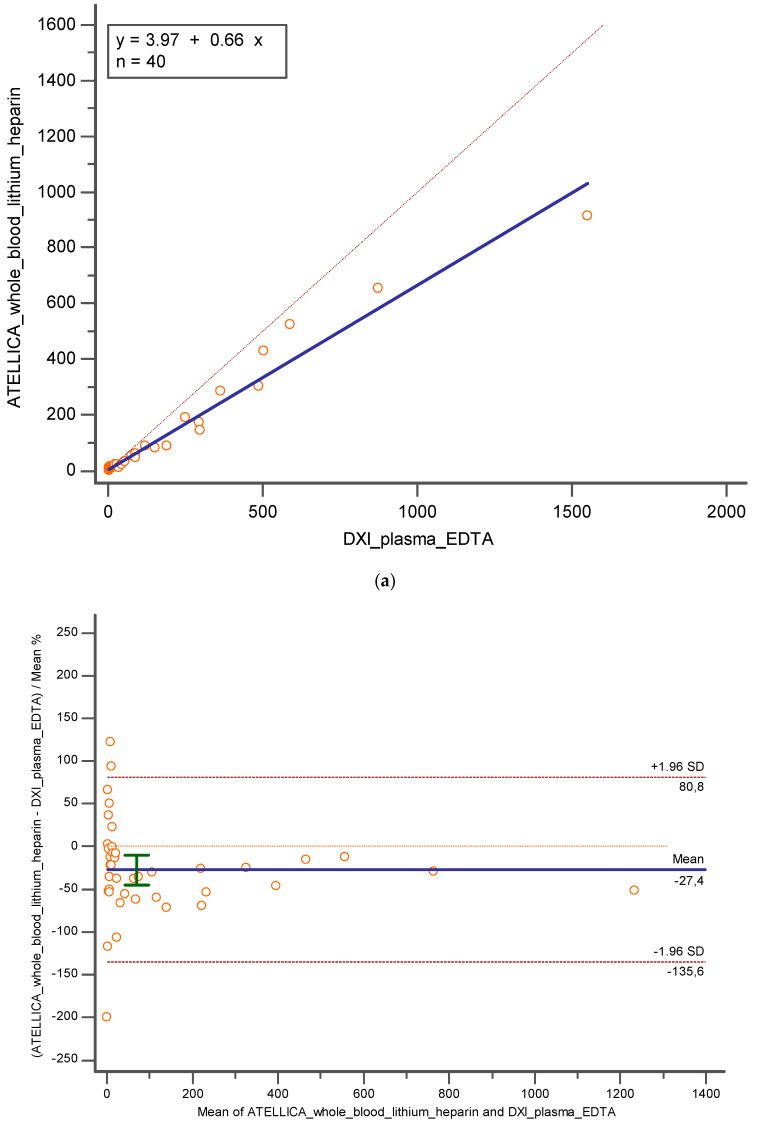

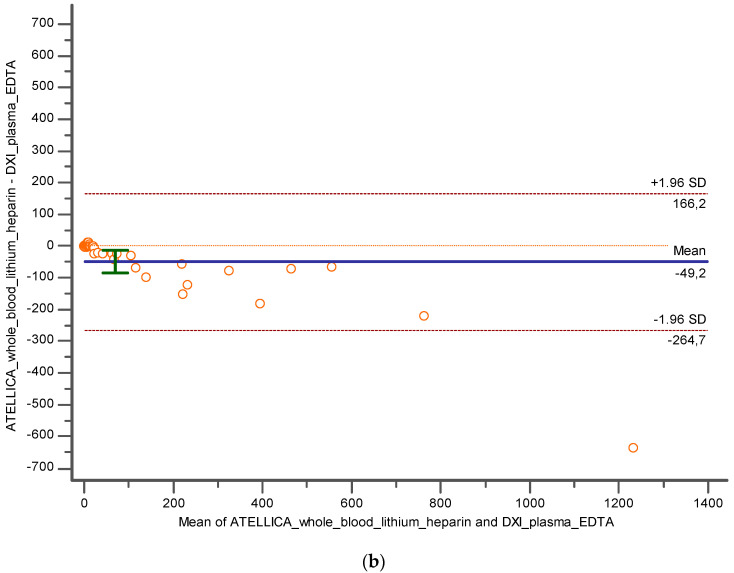
Represents (**a**) Deming regression and (**b**) Bland Altman% between whole blood lithium heparin on Atellica and plasma K_3_EDTA tested on DxI 800. In blue is reported the line of the equation.

Otherwise observing the results between whole blood lithium heparin and plasma K_3_EDTA, a proportional error was present (y = 3.9722 + 0.6602x; slope CI = 95%, [0.4468 to 0.8735]; intercept CI = 95% [−15.54 to 23.48]) and the Pearson correlation coefficient was 0.98 (C = 95%; [0.9671 to 0.9908]), but an underestimation of −27.39% (CI = 95%; [−45.0605 to −9.7389]; *p* < 0.05) and −49.20 (CI = 95%; [−84.3631 to −14.0519]; *p* < 0.05) was still verified (Figure 4a,b).

**Figure 5:**
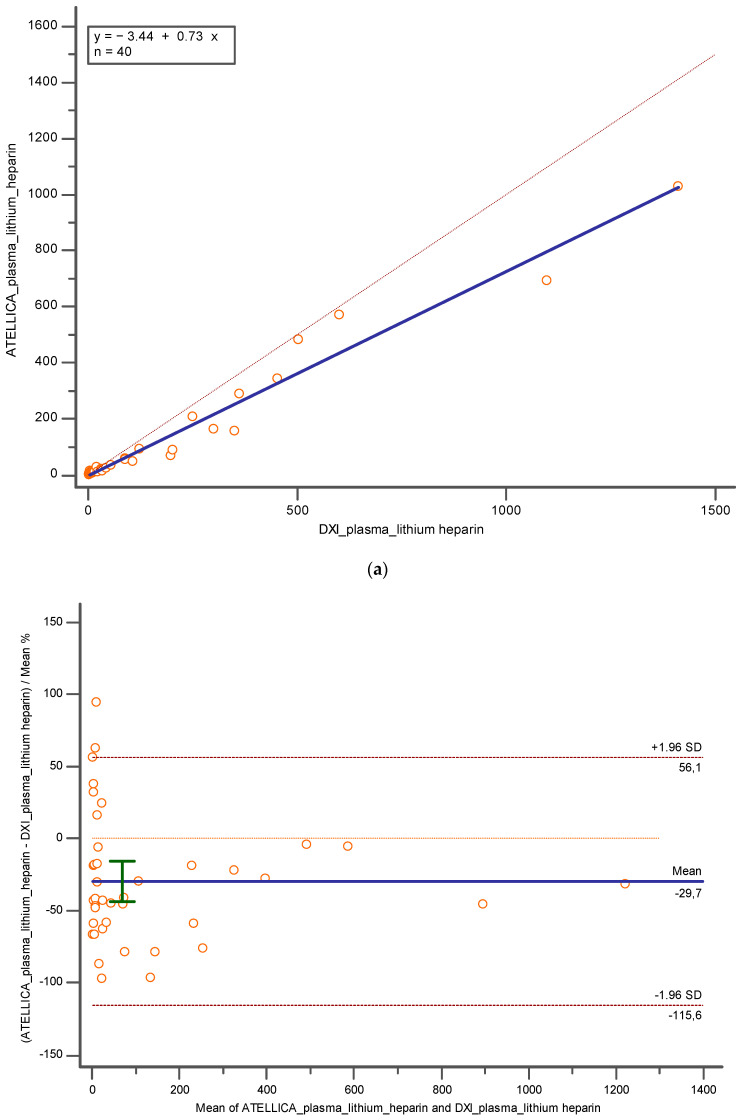

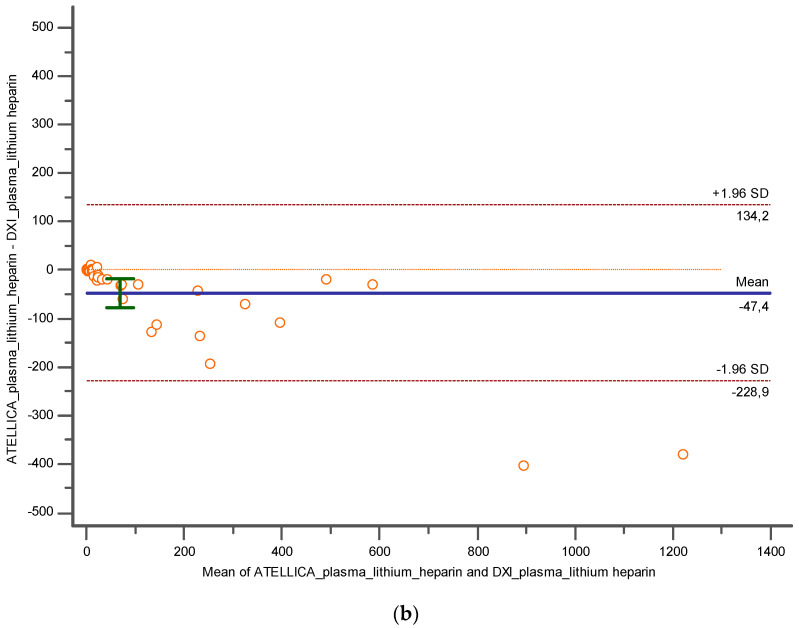
Represents (**a**) Deming regression and (**b**) Bland Altman % comparing lithium heparin plasma employed with Atellica and lithium heparin plasma analyzed with DXI 800. In blue is reported the line of the equation.

Similar results have been observed when taking into account the lithium heparin plasma matrix. When comparing lithium heparin plasma employed with Atellica and lithium heparin plasma analyzed with DxI 800, a proportional error was present (y = −3.4395 + 0.7302x; slope CI = 95%, [0.6395 to 0.8209]; intercept CI = 95% [−12.1131 to 5.2340]), despite a good Pearson correlation coefficient of 0.98 (CI = 95%; [0.9660 to 0.9905]) and no constant error. Also, in this case an underestimation of −29.73% (CI = 95%; [−43.7512 to −15.7281]; *p* < 0.001) and −47.36 (CI = 95%; [−76.9894 to 17.7356]; *p* < 0.05) was detected with Bland Altman (Figure 5a,b).

**Figure 6:**
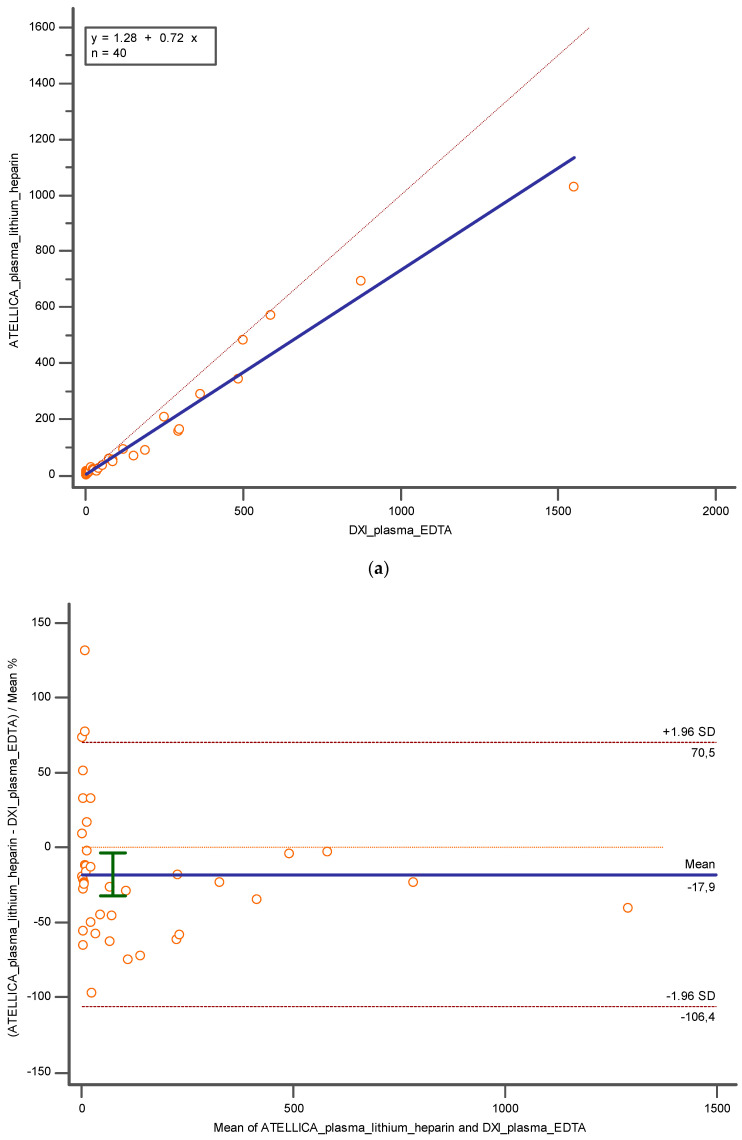

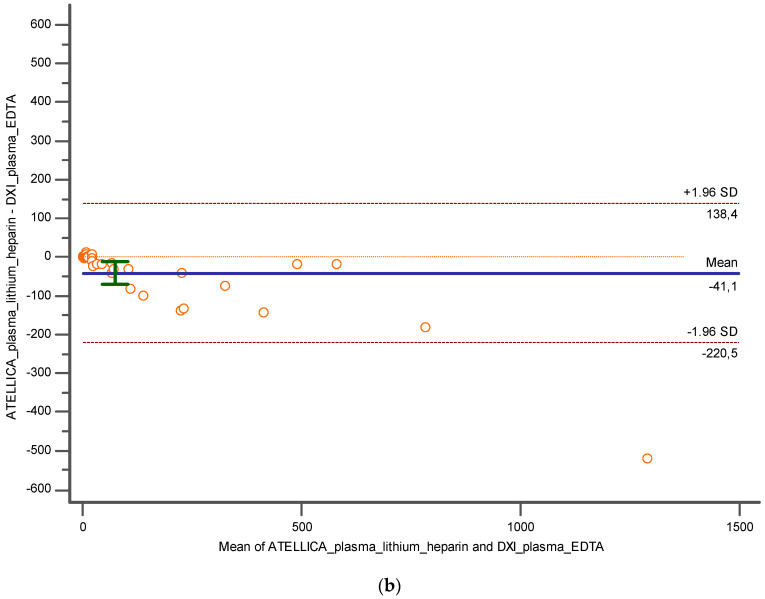
Represents (**a**) Deming regression and (**b**) Bland Altman % between lithium heparin plasma and K3EDTA plasma tested respectively on Atellica and DXI 800. In blue is reported the line of the equation.

Finally, using lithium heparin plasma and K_3_EDTA plasma, still a proportional error was observed (y = 1.2819 + 0.7294x; slope CI = 95%, [0.5339 to 0.9250]; intercept CI = 95% [−16.9704 to 19.5342]) and the Pearson correlation coefficient was confirmed as 0.98 (CI = 95%; [0.97 to 0.99]), but also in this case an underestimation of −17.92% (CI = 95%; [−32.3594 to −3.4973]; *p* < 0.05) and −41.06 (CI = 95%; [−70.3448 to −11.7802]; *p* < 0.05) has been verified with Bland Altman (Figure 6a,b).

Our analysis also considered TnI concentrations close to the cut off of the 99th percentile for our reference population. A pathological TnI is identified when the concentrations are above 10.5 ng/L and 17.8 ng/L among women and men, respectively. A total of 22 samples were used ranging from 1.70 ng/L to 28.30 ng/L. As previously, we investigated the differences between the two different matrices and anticoagulants tested both with Atellica and DxI 800.

**Figure 7:**
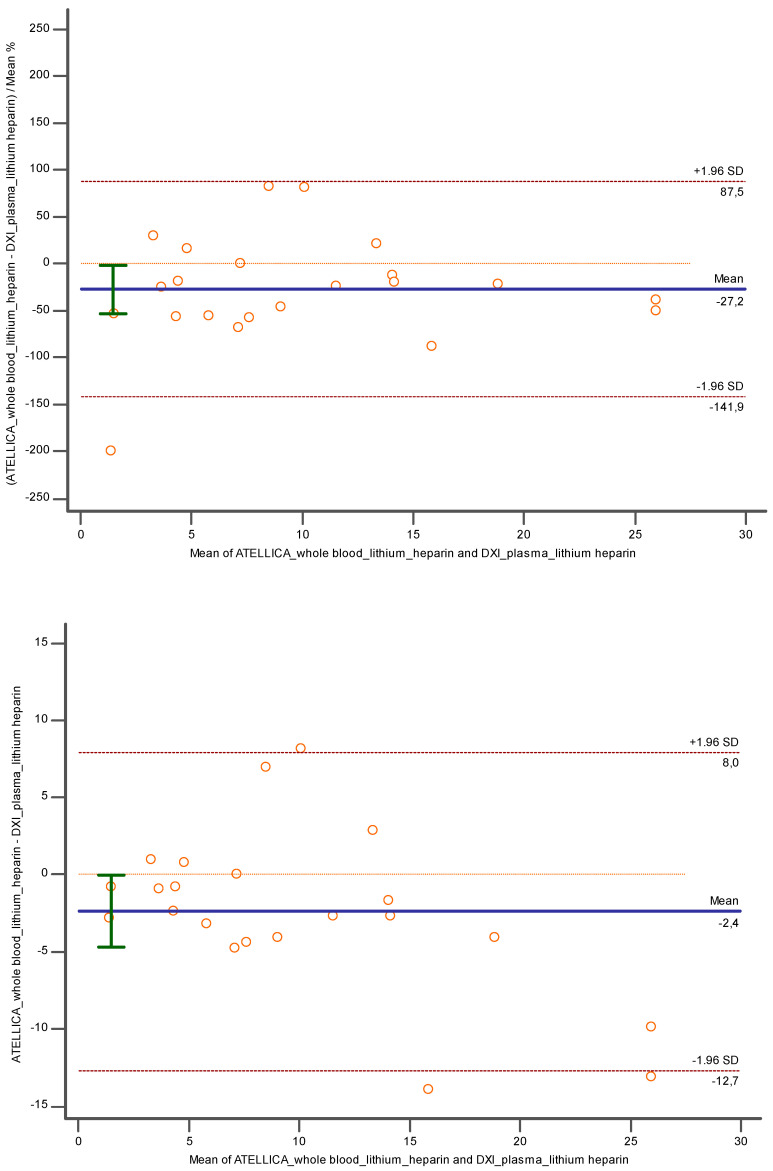
Bland Altman between lithium heparin whole blood and lithium heparin plasma investigated, respectively, with Atellica and DXI 800 close to the related cut off. In blue is reported the line of the equation.

Evaluating the results from lithium heparin whole blood obtained with Atellica versus lithium heparin plasma tested with DxI 800 (Figure 7), a low underestimation of −27.19% (CI = 95%; [−53.14 to −1.25]; *p* < 0.05) and −2.38 (CI = 95%; [−4.71 to −0.04]; *p* < 0.05]) close to the cut off was verified.

**Figure 8:**
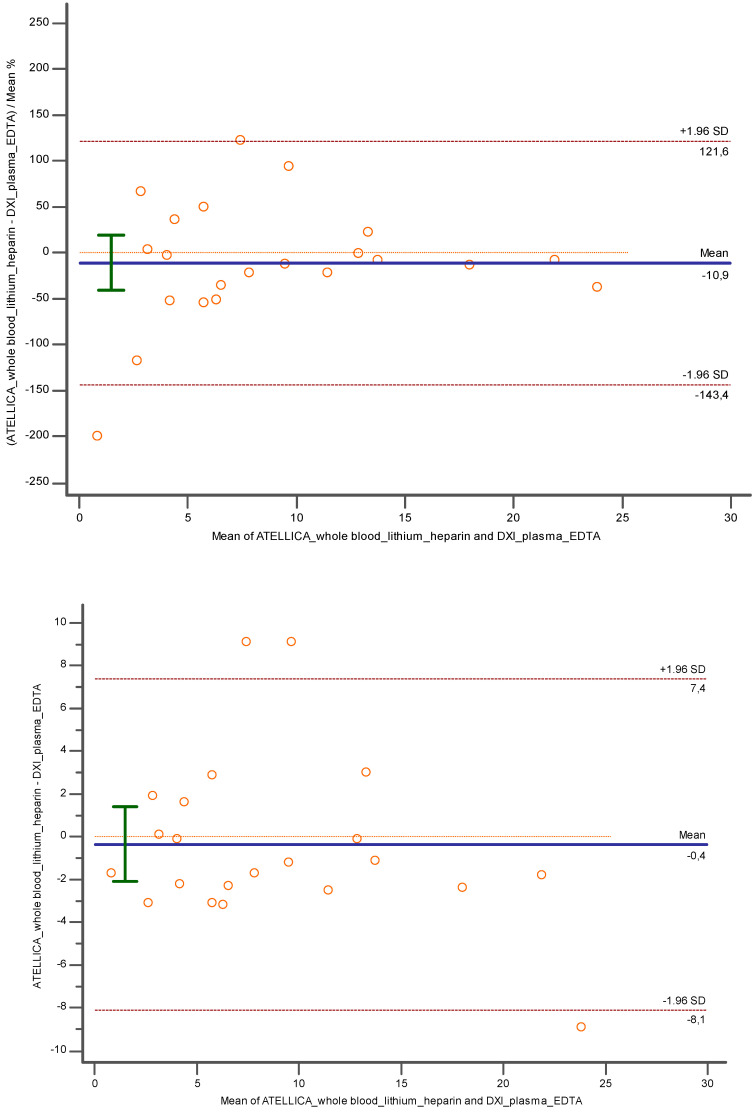
Bland Altman between lithium heparin whole blood and K3EDTA plasma investigated, respectively, with Atellica and DXI 800 close to the related cut off. In blue is reported the line of the equation.

Conversely, considering lithium heparin whole blood and K3EDTA plasma tested, respectively, with Atellica and DxI 800 (Figure 8), a lower bias was encountered: −10.89% (CI = 95%; [−40.87 to 19.07; *p* = 0.45] and −0.35 (CI = 95%; [−2.10 to 1.40]; *p* = 0.68).

**Figure 9:**
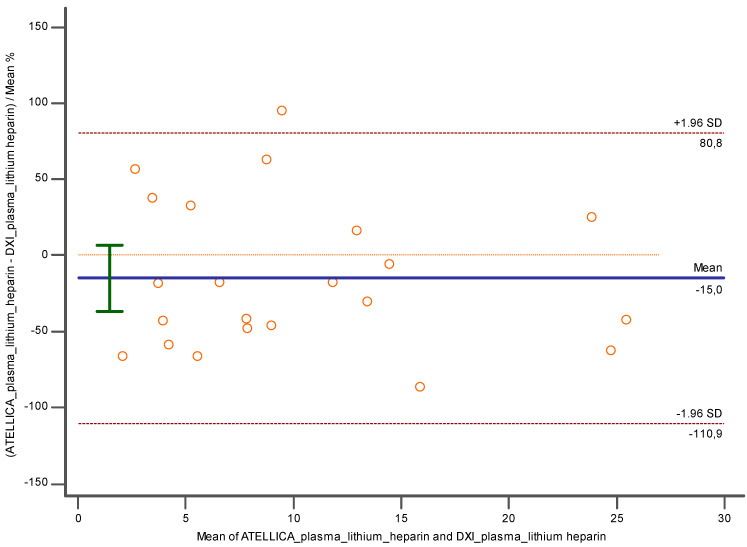

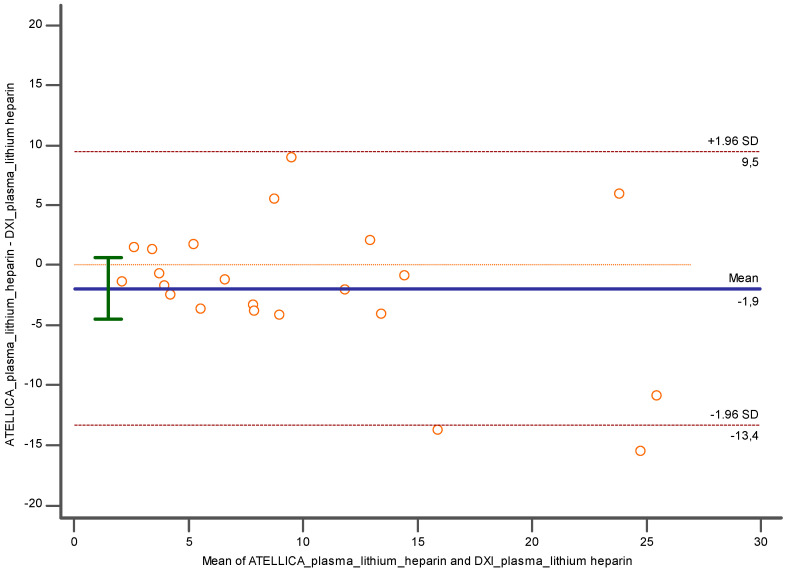
Bland Altman between lithium heparin plasma and lithium heparin plasma investigated, respectively, with Atellica and DXI 800 close to the related cut off. In blue is reported the line of the equation.

Lithium heparin plasma with Atellica versus the same matrix measured with DxI 800 (Figure 9) showed a good performance despite an underestimation of −15% (CI = 95%; [−36.69 to 6.66]; *p* = 0.16) and −1.9 (CI = 95%; [−4.53 to 0.64]; *p* = 0.13).

**Figure 10:**
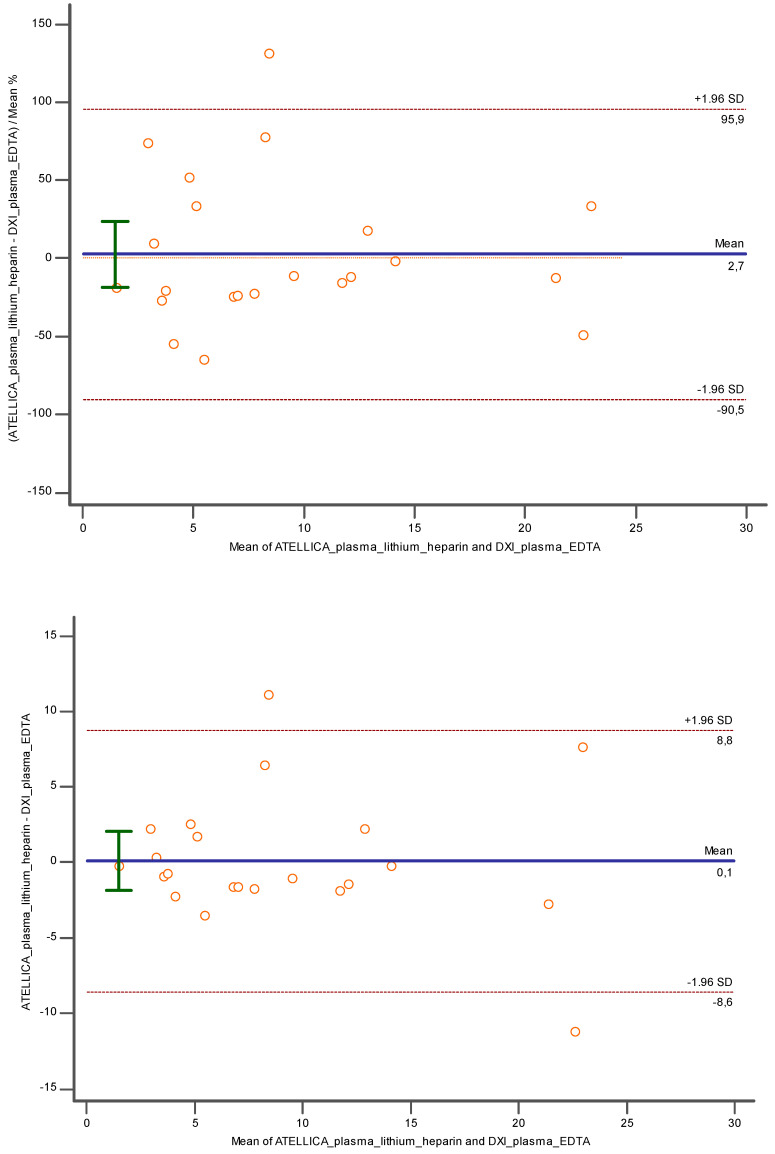
Bland Altman between lithium heparin plasma and K3EDTA plasma investigated, respectively, with Atellica and DXI 800 close to the related cut off. In blue is reported the line of the equation.

Of note is the comparison between plasma lithium heparin and plasma K_3_EDTA. A very good concordance was identified both in percentage (2.7%) and relative values (0.1), demonstrating a possible commutability between these two matrices in TnI evaluation (Figure 10).

## 4. Discussion

Since the COVID-19 pandemic spread in 2020, the importance of rapid results and the possibility to decentralize a group of analyses in an emergency department (ED), emphasized once more the usefulness of POCT technologies. Hengel et al. have highlighted the importance of POCT, especially considering community or care consulting located in difficult geographical areas, where the distance from the first primary care unit takes several minutes to reach [13]. Taking into account the distribution and the elevated number of POCT which can possibly cover a wide area, once more the connectivity is pivotal to guarantee the opportunity of control by the laboratory. The lack of specific guidelines in this regard forced the Emilia Romagna region (Italy) in 2022 to publish a document giving indications for the laboratories working on its territory in order to harmonize the installation and application of these platforms according to the fourth updated edition of ISO 15189:2022 [14].

ISO 15189:2022 regulates also the POCT technologies especially in terms of quality control and governance, giving the laboratory a central role [14]. Once more, the attention was focused on the importance of comparing results of POCT to CL assays, also considering the differences in biological matrices by using different anticoagulants. The choice of whole blood in POCT available in ED is due to the short TAT, since no centrifuge of the sample is necessary. Many Central Laboratory automated analyzers are methods which can use serum, whole blood (Lithium heparin or K_3_EDTA) and lithium heparin, or K_3_EDTA plasma, which need centrifuge. Using different matrices in TnI POCT and CL, instrumentation determination can occur. For example, one could evaluate TnI in ED with POCT for T_0_ and send a sample of plasma lithium heparin to the CL for T_1_h, in order to perform the 0–1 h algorithm. Hence, it is pivotal to investigate any possible bias between POCT and CL instrumentation considering also the biological matrix and the anticoagulant in the exam. These errors could lead physicians to misclassify a diagnosis which could threaten the patient’s health. This issue is once more fundamental when considering that POCT for TnI determination could be available on a paramedical truck, where T_0_ determination could be performed before the patient arrives to the primary care unit.

In addiction, the problem could be even worse when an acute myocardial injury is diagnosed and the patient has to be investigate for myocardial necrosis extension. The TnI is usually performed 48 h after the first episode in order to establish the infarct size. But once more, if bias among matrices is not investigated, confounding variables are able to misclassify the particular situation.

Hence, are those measurements comparable? Can we compare results from different analyzers as POCT and CL instrumentation, especially when different matrices are employed?

Our findings highlight that the opportunity of using different matrices for TnI is possible, but several aspects must be kept in mind. The proportional error found in our comparison is verified when TnI values are increased above the cut off, consisting of an underestimation that could influence the reference change value established by ESC of 50% misclassifying the acute myocardial damages. On the other hand, the differences in TnI concentrations for the patients with a value close to the cut off are not so significant, suggesting that the use of different matrices to exclude a potential ACS condition is potentially possible, especially when plasma K_3_EDTA is used on CL instrumentation. Therefore, considering different concentrations obtained from different analyzers, using different matrices could be potentially useful to exclude significant myocardial damage when the value is below the cut off.

An important limitation of this study is the number of patients enrolled, which is the minimum recommend by the CLSI EP-09. Increasing the number of cases and the opportunity to evaluate a distribution the of concentrations covering a wider range could ensure the robustness of our claim.

## 5. Conclusions

Point of care technologies could be considered the future of emergency care diagnostics and beyond. A possible scenario could be the opportunity to have POCT also in conditions where a short TAT is crucial. For example, perioperative procedures where measures of specific analytes are critical in order to evaluate possible damage caused by the surgery.

Furthermore, the use of capillary blood and the consumption of very small volumes of sample is also suitable for caregiving centers, limiting the distress of blood sampling and encouraging patients to undergo to periodical controls.

However, different considerations are pivotal. First of all, we must always ensure the comparability of measures performed with a POCT versus CL instruments. Second, all pre-analytical aspects (for example, the correct anticoagulant choice) must be known and respected by all users. Basically, the average operators of POCT are personnel not so confident with laboratory instrumentation or laboratory quality politics and these technologies are deployed in a department where no laboratory technicians or physicians are present. The laboratory has a central responsibility regarding these issues and must provide continuous training of the operators involved in the use of POCT through workshops, e-learning, and webinars, in order to educate new figures in the use of POCT and to discuss case reports or topics encountered during the daily use of POCT to secure a continuous update of knowledge.

Another important aspect for POCT is the importance of quality controls and an external proficiency test (EPT) which are finalized to verify the accuracy of the measurements. This is a very tough argument, since nowadays the lack of commutable materials for different analytes, the difficulty to provide stable controls, and the low number of participants to EPT make it hard for a laboratory to evaluate its performance as usual.

The evolution of troponin assays continues, and POCT hs-cTn assays soon will become more widely accessible. Evidence is required to ensure that emerging POCT hs-cTn assays meet both analytical and clinical needs, and a robust redesign of models of care will be needed to maximize the potential benefits.

In conclusion, since the POCT TnI has the potential to be the very first choice of evaluation in suspected ACS patient settings, we suggest using the same method, matrix, and anticoagulant in 0–1 h algorithm.

## Figures and Tables

**Table 1:** Performance characteristics of troponin assays.

Platform	Company	Concentration at 10% CV	Specimen Type	99th Percentile	Percent Normals Measured ≥ LoD	Assay Type/Device	Sample Volume	Assay Time
Atellica VTLi	Siemens, Bayern München, Germany	NP (20% CV 6.7 ng/L)	Capillary, WB and plasma Li-heparin	Overall: 23 ng/L F: 18 ng/L M: 27 ng/L	Overall: 83.7% F: 79.7% M: 87.3%	Immunoassay; cds	50 μL	<8 min
DxI 800	Beckman Coulter	10% CV 5.6 ng/L	Serum, Plasma Li-heparin and K_3_EDTA	Overall: 17.5 ng/L F: 11.6 ng/L M: 19.8 ng/L	Overall: >50%	chemiluminescent immunoassay	100 μL	18 min

cds, compact desktop systems; CV, coefficient of variation; Li, lithium; NP, not provided; WB, whole blood; LoD, limit of detection.

## Data Availability

No new data were created or analyzed in this study. Data sharing is not applicable to this article.

## References

[1] Wu A.H.B., Christenson R.H., Greene D.N., Jaffe A.S., Kavsak P.A., Ordonez-Llanos J., Apple F.S. (2018). Clinical laboratory practice recommendations for the use of cardiac troponin in acute coronary syndrome: Expert opinion from the Academy of the American Association for Clinical Chemistry and the Task Force on Clinical Applications of Cardiac Bio-Markers of the International Federation of Clinical Chemistry and Laboratory Medicine. Clin. Chem..

[2] Collet J.P., Thiele H., Barbato E., Barthélémy O., Bauersachs J., Bhatt D.L., Dendale P., Dorobantu M., Edvardsen T., Folliguet T. (2021). 2020 ESC Guidelines for the management of acute coronary syndromes in patients presenting without persistent ST-segment elevation. Eur. Heart J..

[3] Collinson P.O., Saenger A.K., Apple F.S. (2019). IFCCC-CB High sensitivity, contemporary and point-of-care cardiac troponin assays: Educational aids developed by the IFCC Committee on Clinical Application of Cardiac Bio-Markers. Clin. Chem. Lab. Med..

[4] Vafaie M., Biener M., Mueller M., Abu Sharar H., Hartmann O., Hertel S., Katus H.A., Giannitsis E. (2015). Addition of copeptin improves diagnostic performance of point-of-care testing (POCT) for cardiac troponin T in early rule-out of myocardial infarction—A pilot study. Int. J. Cardiol..

[5] Clerico A., Zaninotto M., Plebani M. (2021). High-sensitivity assay for cardiac troponins with POCT methods. The future is soon. Clin. Chem. Lab. Med..

[6] Sörensen N.A., Neumann J.T., Ojeda F., Giannitsis E., Spanuth E., Blankenberg S., Westermann D., Zeller T. (2019). Diagnostic evaluation of a high-sensitivity troponin I point-of-care assay. Clin. Chem..

[7] Boeddinghaus J., Nestelberger T., Koechlin L., Wussler D., Lopez-Ayala P., Walter J.E., Troester V., Ratmann P.D., Seidel F., Zimmermann T. (2020). Early diagnosis of myocardial infarction with Point-of-Care high-sensitivity cardiac troponin I. J. Am. Coll. Cardiol..

[8] Apple F.S., Schulz K., Schmidt C.W., van Domburg T.S.Y., Fonville J.M., de Theije F.K. (2021). Determination of sex-specific 99th percentile upper reference limits for a point of care high sensitivity cardiac troponin I assay. Clin. Chem. Lab. Med..

[9] Christenson R.H., Frenk L.D.S., de Graaf H.J., van Domburg T.S.Y., Wijnands F.P.G., Foolen H.W.J., Kemper D.W.M., Bruinen A.L., Meijering B.D.M., Fonville J.M. (2022). Point-of-Care: Roadmap for Analytical Characterization and Validation of a High-Sensitivity Cardiac Troponin I Assay in Plasma and Whole Blood Matrices. J. Appl. Lab. Med..

[10] Clinical and Laboratory Standards Institute (CLSI) (2018). Measurement procedure comparison and bias estimation using patient samples. Approved Guideline.

[11] Lippi G., Ferrari A., Gandini G., Gelati M., Lo Cascio C., Salvagno G.L. (2017). Analytical evaluation of the new Beckman Coulter Access high sensitivity cardiac troponin I immunoassay. Clin. Chem. Lab. Med..

[12] Bruls D.M., Evers T.H., Kahlman J.A.H., van Lankvelt P.J.W., Ovsyanko M., Pelssers E.G.M., Schleipen J.J.H.B., de Theije F.K., Verschuren C.A., van der Wijk T. (2009). Rapid integrated biosensor for multiplexed immunoassays based on actuated magnetic nanoparticles. Lab Chip.

[13] Hengel B., Causer L., Matthews S., Smith K., Andrewartha K., Badman S., Spaeth B., Tangey A., Cunningham P., Saha A. (2021). A decentralised point-of-care testing model to address inequities in the COVID-19 response. Lancet Infect. Dis..

[14] (2022). Medical Laboratories—Requirements for Quality and Competence.

